# Atomistic Insights into Activation and Degradation of La_0.6_Sr_0.4_CoO_3−δ_ Electrocatalysts under Oxygen Evolution Conditions

**DOI:** 10.1021/jacs.2c07226

**Published:** 2022-09-21

**Authors:** Moritz L. Weber, Gaurav Lole, Attila Kormanyos, Alexander Schwiers, Lisa Heymann, Florian D. Speck, Tobias Meyer, Regina Dittmann, Serhiy Cherevko, Christian Jooss, Christoph Baeumer, Felix Gunkel

**Affiliations:** † Peter Grünberg Institute (PGI-7) and Jülich-Aachen Research Alliance (JARA-FIT), Forschungszentrum Jülich GmbH, Jülich 52425, Germany; ‡ Institute of Materials Physics, University of Göttingen, Göttingen 37077, Germany; § International Center for Advanced Studies of Energy Conversion (ICASEC), University of Göttingen, Göttingen 37077, Germany; ∥ Helmholtz-Institute Erlangen-Nürnberg for Renewable Energy (IEK-11), Forschungszentrum Jülich GmbH, Erlangen 91058, Germany; ⊥ Department of Chemical and Biological Engineering, Friedrich-Alexander-Universität Erlangen-Nürnberg, Erlangen 91058, Germany; # Faculty of Science and Technology, MESA+ Institute for Nanotechnology, University of Twente, Enschede 7500 AE, The Netherlands

## Abstract

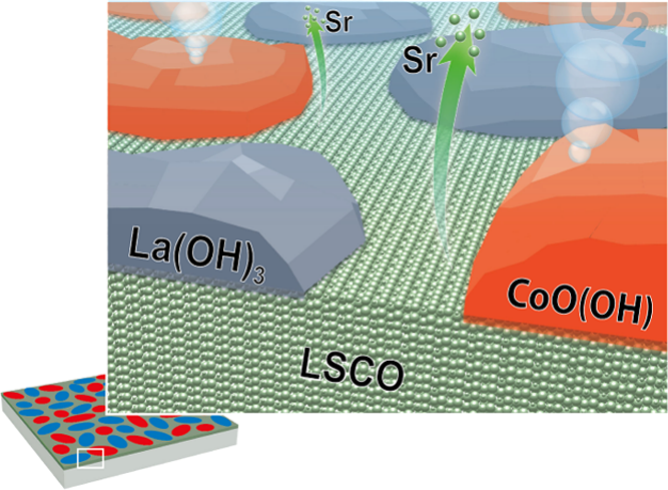

The stability of perovskite oxide catalysts for the oxygen evolution reaction (OER) plays a critical role in their applicability in water splitting concepts. Decomposition of perovskite oxides under applied potential is typically linked to cation leaching and amorphization of the material. However, structural changes and phase transformations at the catalyst surface were also shown to govern the activity of several perovskite electrocatalysts under applied potential. Hence, it is crucial for the rational design of durable perovskite catalysts to understand the interplay between the formation of active surface phases and stability limitations under OER conditions. In the present study, we reveal a surface-dominated activation and deactivation mechanism of the prominent electrocatalyst La_0.6_Sr_0.4_CoO_3−δ_ under steady-state OER conditions. Using a multiscale microscopy and spectroscopy approach, we identify the evolving Co-oxyhydroxide as catalytically active surface species and La-hydroxide as inactive species involved in the transient degradation behavior of the catalyst. While the leaching of Sr results in the formation of mixed surface phases, which can be considered as a part of the active surface, the gradual depletion of Co from a self-assembled active CoO(OH) phase and the relative enrichment of passivating La(OH)_3_ at the electrode surface result in the failure of the perovskite catalyst under applied potential.

## Introduction

Active and durable energy materials are key to establish a sustainable energy management based on efficient devices for the production, conversion, and storage of chemical fuels based on renewable energy, much needed to abandon climate-damaging fossil fuels.^1−3^ Essentially, the sluggish oxygen evolution reaction (OER) thwarts the implementation of water splitting concepts for the production of hydrogen. To overcome this limitation, the exceptional catalytic activity of perovskite oxides for the OER has stimulated the discussion about the intrinsic properties responsible for the high catalytic performance of this material class for many years.^4−7^ Here, the attention increasingly lies on the processes at the topmost surface and in the near-surface region of the oxide catalysts, which influence the catalytic activity of perovskite materials.^8−12^ In addition to activity, the stability of perovskite electrocatalysts in alkaline media and under applied potential is highly debated since high robustness is crucial for their applicability in energy devices.^4,10,11,13,14^ Numerous studies have indicated that electrocatalysts undergo surface reconstructions and chemical transformations under OER conditions that may be related to the transformation of the perovskite surface toward an active, dynamic state and also to detrimental processes leading to irreversible degradation. Here, leaching of cations^11,15−20^ as well as structural modifications such as amorphization^11,15,17,18,21−25^ and even complete decomposition of oxide catalysts^26,27^ were observed and linked to changes in the catalysts’ activity. At the same time, recent studies have shown that near-surface phase transitions may play a key role in the catalytic process.^8,12,28^

Hence, it is apparent that surface phase transformations can trigger catalytic activity and are also involved in the aging of catalysts. The link between these processes however remains unresolved to date. Therefore, a holistic view on both activity and stability of perovskite OER catalysts, with respect to dynamic surface processes involved in the oxygen evolution reaction, is needed to develop strategies to overcome the stability limitations of active perovskite electrocatalysts. Epitaxial model catalysts offer a high level of control in material properties and thus attracted much attention for the study of catalytic processes at well-defined perovskite surfaces.^11,12,14,26,29−32^ In the present study, we identify active species at the surface of LSCO at the atomic scale and provide a detailed understanding of the potential-driven, dynamic processes at the solid–liquid interface under OER conditions that result in the formation of Co-oxyhydroxide as active surface species and La-hydroxide as inactive passivation layer. Our findings link the transformation of the crystalline perovskite catalyst toward mixed chemical phases in the near-surface region to the degradation behavior of LSCO during operation in alkaline media.

## Results

### Catalyst Efficacy and Lifetime at Increased OER Reaction Rates

**Figure 1:**
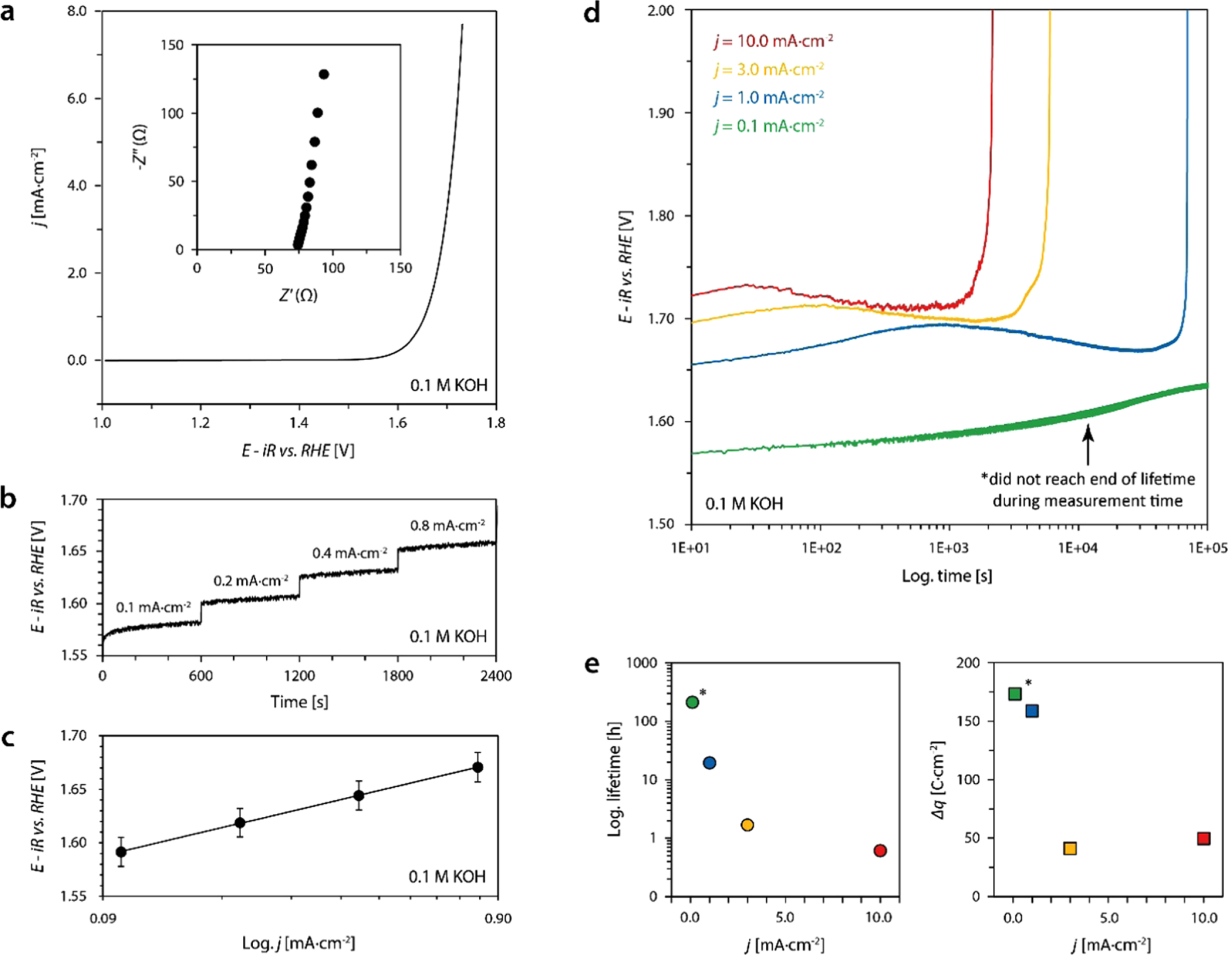
Electrochemical performance of 20 nm epitaxial (001) LSCO thin films catalyzing the oxygen evolution reaction (OER). (a) Averaged forward and reverse scans of the second cycle of cyclic voltammetry measurements; the inset image shows the Nyquist plot obtained by electrochemical impedance spectroscopy. (b) Steady-state measurement of four consecutive galvanostatic holds. (c) Tafel plot obtained from steady-state galvanostatic measurements; error bars represent the standard deviation of average values obtained by the measurement of three different samples. (d) Chronopotentiometric measurements for the characterization of the LSCO stability on the basis of the catalyst lifetimes at different applied current densities. Abrupt increase of potential denotes the end of lifetime. (e) Plot of the lifetimes (left) and the respective transferred charge (right) depending on the applied current density determined based on the data shown in panel (d), visualizing a strong nonlinearity, i.e., potential dependence of the catalyst deactivation.

The electrochemical performance of 20-nm-thick epitaxial LSCO electrocatalysts was characterized in 0.1 M KOH using a rotating disk setup. Details on the sample preparation can be found in the Experimental Section and ref (26). Figure 1a shows representative cyclic voltammetry data, where an *iR*-corrected potential of *E* = 1.66 ± 0.01 V vs RHE was determined at a current density of *j* = 1.0 mA·cm^–2^ based on the average value obtained from three different samples. A representative Nyquist plot, obtained by electrochemical impedance spectroscopy (EIS) at open-circuit potential is shown as an inset image. Furthermore, Tafel analysis was performed by consecutive steady-state galvanostatic holds at different current densities as displayed in Figure 1b. The Tafel plot derived from averaged values obtained from three samples is given in Figure 1c with a Tafel slope of ∂*V*/∂log(*j*) ∼88 mV/dec, which is in good agreement with the literature.^33,34^ All LSCO layers hence show high OER activity comparable to the best-in-class perovskite catalyst.

To evaluate the stability, i.e., the lifetime, of the LSCO electrocatalysts during OER operation, chronopotentiometry was performed at different current densities. Here, the oxygen evolution reaction is driven under steady-state conditions (constant reaction rate) until the deactivation of the LSCO thin-film electrodes is evident from a rapid increase in overpotential. All samples used for lifetime testing experienced equal electrochemical treatment before the respective galvanostatic measurements. A detailed description of the measurement protocol is given in the Experimental Section. The end of lifetime represents a sudden loss of the OER activity. Increasing lifetimes are observed for decreasing current densities, ranging between 0.6 and 19.5 h in the current density range between *j* = 1.0 and 10.0 mA·cm^–2^ (Figure 1d). Notably, the lifetime measurement of epitaxial LSCO at *j* = 0.1 mA·cm^–2^ remained stable for >200 h and did not reach the end of its lifetime during the measurement time (denoted by asterisk). The investigation of the potential-dependent catalyst lifetime reveals substantial, though considerably varying stability of the epitaxial thin-film electrodes of only 20 nm thickness. As can be seen, the lifetime shows a pronounced nonlinear trend for LSCO catalysts operated at different current densities (Figure 1e, left). Similarly, the total charge Δ*q*, indicative of the total amount of oxygen generated during the catalyst life, strongly depends on the applied potential (Figure 1e, right). This results in pronounced differences in the efficiency of the catalysts depending on the operation conditions, where low reaction rates at the catalyst surface lead to higher stability, while high current densities and high potentials result in rapid catalyst deactivation. Consequently, dynamic processes need to be considered to gain fundamental understanding of the degradation behavior of LSCO electrocatalysts.

### Surface-Dominated Catalyst Deactivation under Steady-State OER Operation

**Figure 2:**
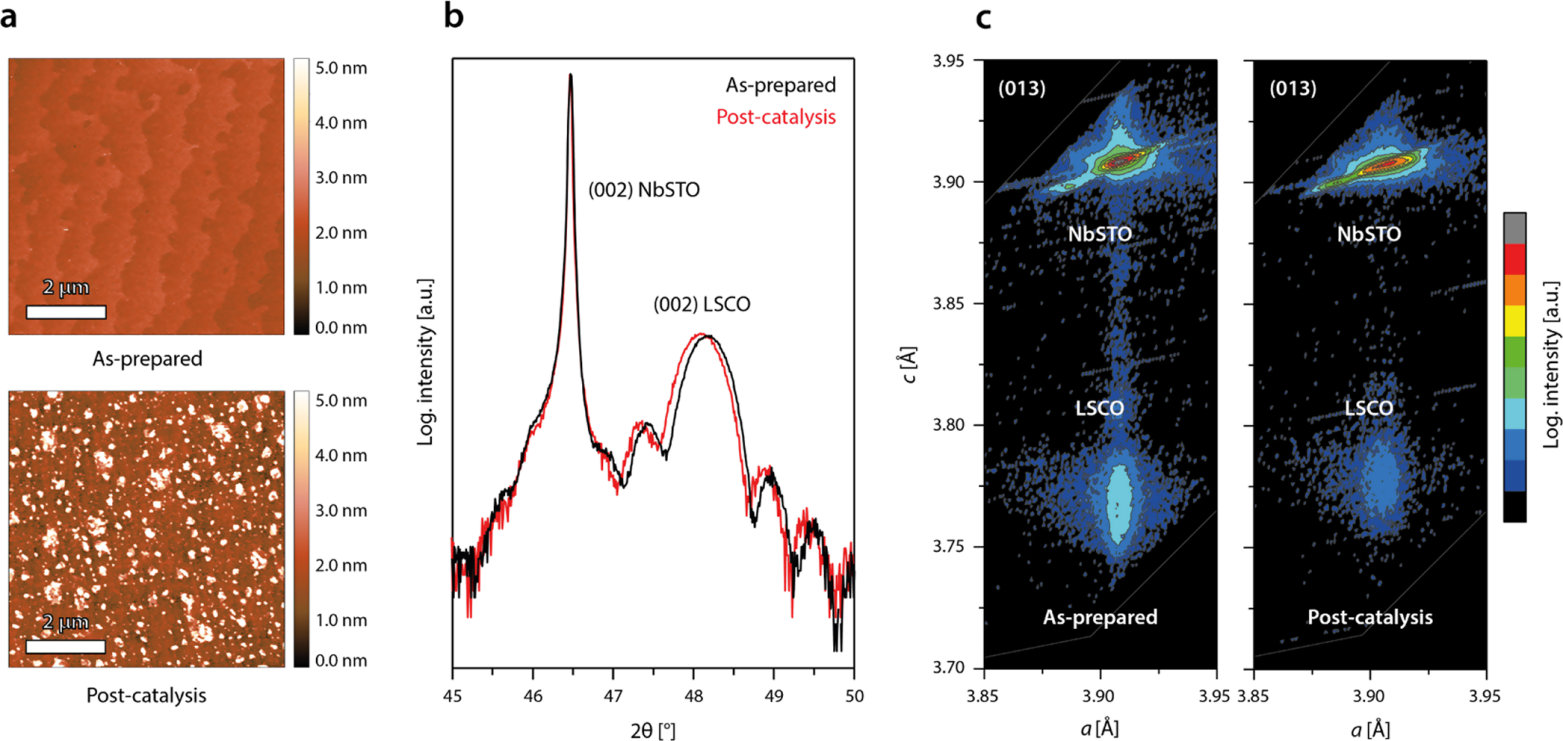
(a) Representative atomic force microscopy (AFM) images of an LSCO thin film comparing the morphology in the as-prepared state and after the end of lifetime (operated at *j* = 10.0 mA·cm^–2^). (b) X-ray diffraction 2θ–ω analysis reveals a high crystallinity and similar thickness of the catalyst layers in the as-prepared and post-catalysis state. (c) X-ray diffraction in reciprocal space mapping geometry around the asymmetric (013) reflections confirms minor expansion in the thin-film *c*-lattice parameter, while epitaxial strain is found to be preserved after electrochemical operation (constant *a*-lattice parameter).

After steady-state operation of the epitaxial LSCO model electrodes until the end of lifetime, the formation of island-like structures is visible at the initially smooth surface by atomic force microscopy (AFM), which exhibits a distinct step terrace structure in the pristine state (Figure 2a). In spite of the severe morphologic changes, X-ray diffraction analysis (XRD) in 2θ–ω measurement geometry reveals only minor changes in the bulk properties of the thin-film catalysts, reflected by a slight broadening of the thin-film diffraction peak and a slight shift of the peak position toward lower diffraction angles (Figure 2b). Reciprocal space mapping confirms a slight expansion of the *c*-lattice parameter, while the strain state is preserved as evident from the constant *a*-lattice parameter (Figure 2c).

These rather marginal changes in the bulk properties, however, are unlikely to explain the complete deactivation of the catalysts. In fact, they demonstrate the high dissolution stability of the epitaxial thin-film catalysts in steady-state operation mode. Nevertheless, a slight decrease in thickness of the crystalline LSCO layer can be observed from the XRD analysis that indicates a loss of the structural order of the perovskite material in the near-surface region. The degradation zone can be estimated to be about ∼1 nm in depth for thin-film electrodes operated at high current densities of *j* = 10.0 mA·cm^–2^ based on the periodicity of the thickness oscillations. After operation at lower current densities, the degradation depth was found to be slightly increased to several nanometers, which is consistent with an increased amount of transferred charge in the low potential regime (cf. Figure 1e). The lifetime of LSCO electrocatalysts under steady-state operation at increased current densities, therefore, might be rather determined by a surface-dominated deactivation mechanism than a bulk process.

In contrast, bulk degradation and amorphization of the entire catalytic thin films were reported under repeated dynamic cycling.^26^ The decreased amorphization rate under steady-state operation conditions in comparison to dynamic operation conditions during commonly applied potential cycling like cyclic voltammetry is consistent with the observations of May et al.^15^ However, the limitation of structural changes to the topmost surface during steady-state operation at relatively increased current densities associated with the accelerated failure of the electrocatalysts is surprising.

### Surface Mobility of Cobalt Moieties Promotes Activity and Surface Disorder

**Figure 3:**
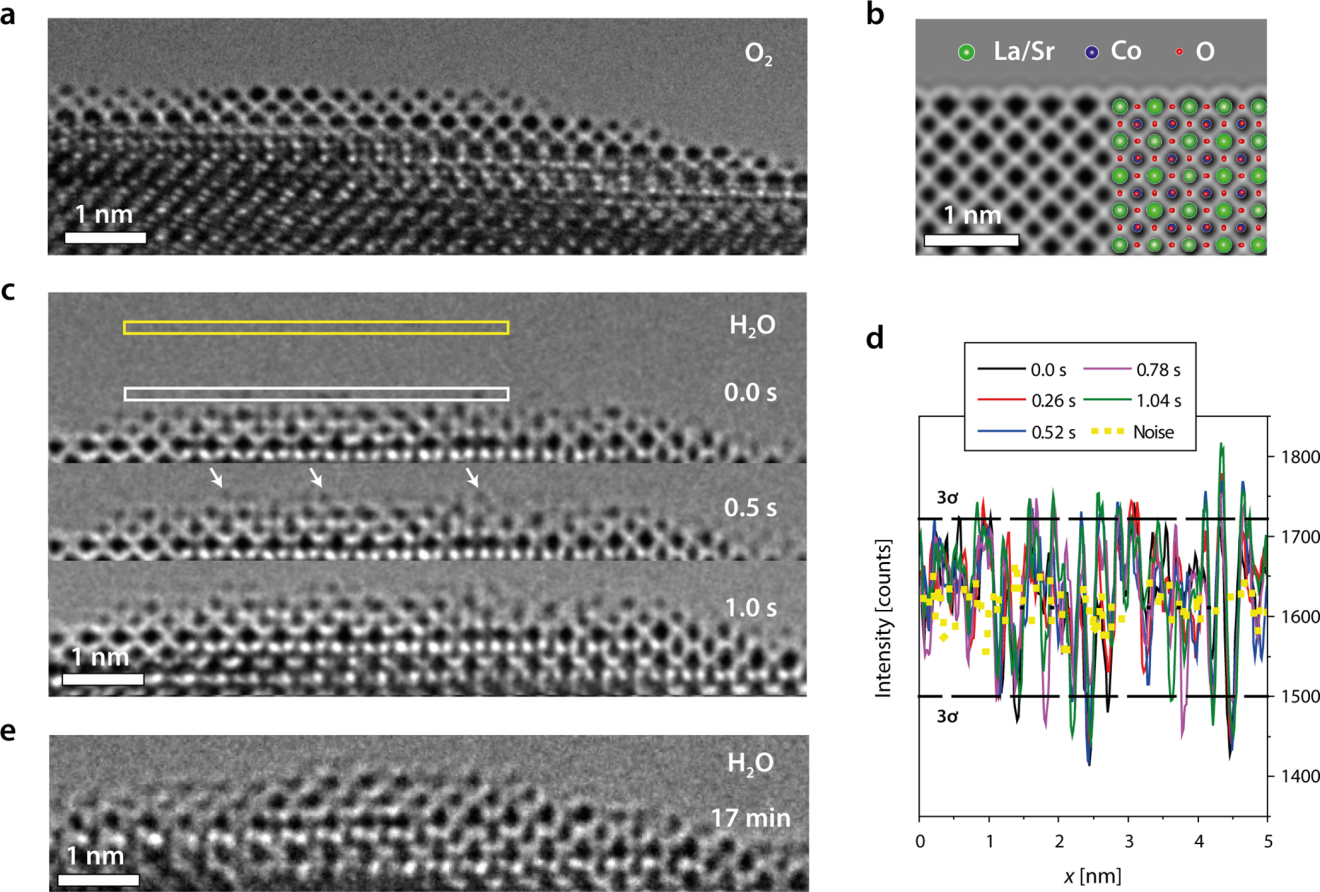
Observation of the dynamic LSCO (001) surface by ETEM. (a) Surface of the as-prepared LSCO lamella is atomically sharp and exhibits A-site termination in O_2_ environment. (b) Simulated image of the LSCO (001) surface shows atomic contrast that is consistent with the experimentally obtained HRTEM image. A superimposed structural model illustrates the atomic positions of Sr, La, Co, and O. (c) Representative images of a time sequence (4 fps) demonstrate the LSCO surface dynamics in 0.5 Pa of H_2_O. Highly mobile adatoms are detected at the solid–liquid interface under near-OER conditions, which appear at the B-site positions of the extended perovskite surface. White arrows highlight several exemplary Co adatoms. (d) Line profiles are recorded close above the A-site-terminated surface as indicated by the white rectangle in panel (c) to quantitatively evaluate the hopping events. The noise level is determined above the sample (yellow rectangle) and the 3σ threshold is calculated from the noise signal, which indicates the detection limit of Co adatoms. Intensity fluctuations above 3σ level indicate the presence of highly mobile adatoms at the interface between the electrocatalysts and the condensed water layer. (e) Structural transformation of the crystalline LSCO surface region toward a disordered layer is detected under near-OER conditions. The image was recorded after imaging under anodic polarization for 17 min in 7 Pa of H_2_O.

To elucidate the atomistic details of the catalytic OER process, we investigated the LSCO samples under near-OER conditions using environmental transmission electron microscopy (ETEM), which allows to study the catalyst surface in direct contact to adsorbed water. The experimental details and the lamella preparation procedure followed the routines established in references^35,36^ and are described in the Experimental Section. After a recrystallization procedure of the FIB lamella in O_2_ atmosphere (cf. Figure S1), an atomically sharp surface is obtained, ideal for atomic-resolution studies of surface processes at the solid–liquid interface (Figure 3a). The surface is well-ordered and exhibits a sharp A-site termination, which remains stable in O_2_ environment (cf. Figure S2 and Movie M1). Here, the experimentally observed atomic ordering is well-comparable to a simulated image of the LSCO surface structure (Figure 3b), which indicates a predominant A-site termination of the as-prepared (001) perovskite electrocatalyst, consistent with a typically observed A-site-enriched surface. To investigate the LSCO surface structure under near-OER conditions, the imaging environment is switched from O_2_ to H_2_O conditions, which results in the condensation of a thin water layer at the perovskite surface. In addition, the incident electron beam induces an anodic potential to the sample, leading to effective near-OER conditions. A time sequence of HR-ETEM images of the interface recorded in 0.5 Pa of H_2_O is shown in Figure 3c (cf. Movie M2), which reveals dynamical changes of the surface structure that occur within the timespan of seconds to minutes after exposure to the water environment. Here, the presence of dynamic adatoms on top of the ordered A-site-terminated surface is observed (white arrows at *t* = 0.5 s). Based on the emerging contrast and their location at specific B-sites of the extended perovskite lattice, they are attributed as Co species. They can form dynamic active sites at the solid–liquid interface under anodic polarization of the OER catalyst. This observation is consistent with sweep rate-dependent cyclic voltammetry that reveals an increase in the magnitude of the exchange current for (operated, but still active) LSCO electrocatalysts after operation under OER conditions relative to the as-prepared state, which is accompanied by more pronounced redox features (cf. Figure S3). As these ionic processes occur on very short time scales in comparison to the catalyst lifetimes they are likely to play a key role in the catalyst activity. At the same time, they might affect the atomistic processes responsible for catalyst degradation.

The high mobility of the adatoms is further illustrated by line profiles shown in Figure 3d, extracted right above the topmost A-site column at the solid–liquid interface. The background fluctuations of the CCD signal are recorded in the vacuum region above the surface as a reference and yield the standard deviation 3σ, which defines the noise level of the measurements. Each signal above the 3σ-level hence indicates the appearance and disappearance of dynamic Co moieties at the catalyst surface. The hopping rate, i.e., the presence of active Co species at the LSCO surface was detected to be increased in H_2_O environment by a factor of eight (*r*(O_2_) ∼ 0.5 s^–1^ vs *r*(H_2_O) ∼ 4.0 s^–1^, cf. Figure S2). The increased mobility of Co cations results in transient changes of the surface structure, indicative of the degradation process associated with the evolution of the initially atomically sharp surface toward a disordered and Co-rich surface layer at the catalyst–electrolyte interface, as can be seen in Figure 3e (cf. Movie M3). The surface reconstruction that includes displacements and mixing of A- and B-site cations along the disordered surface was observed to be accelerated by higher partial pressures of H_2_O. Consistently, imaging in 0.5 Pa of H_2_O results in a slow formation of a disordered surface layer (Figure S4a), while at 11 Pa of H_2_O, a fast formation of the disordered surface is observed (Figure S4b and Movie M4). Here, the disordered surface layer exhibits a thickness of about ∼1 nm, well-comparable to the degradation zone detected by XRD after OER operation at high current densities (cf. Figure 2b,c).

**Figure 4:**
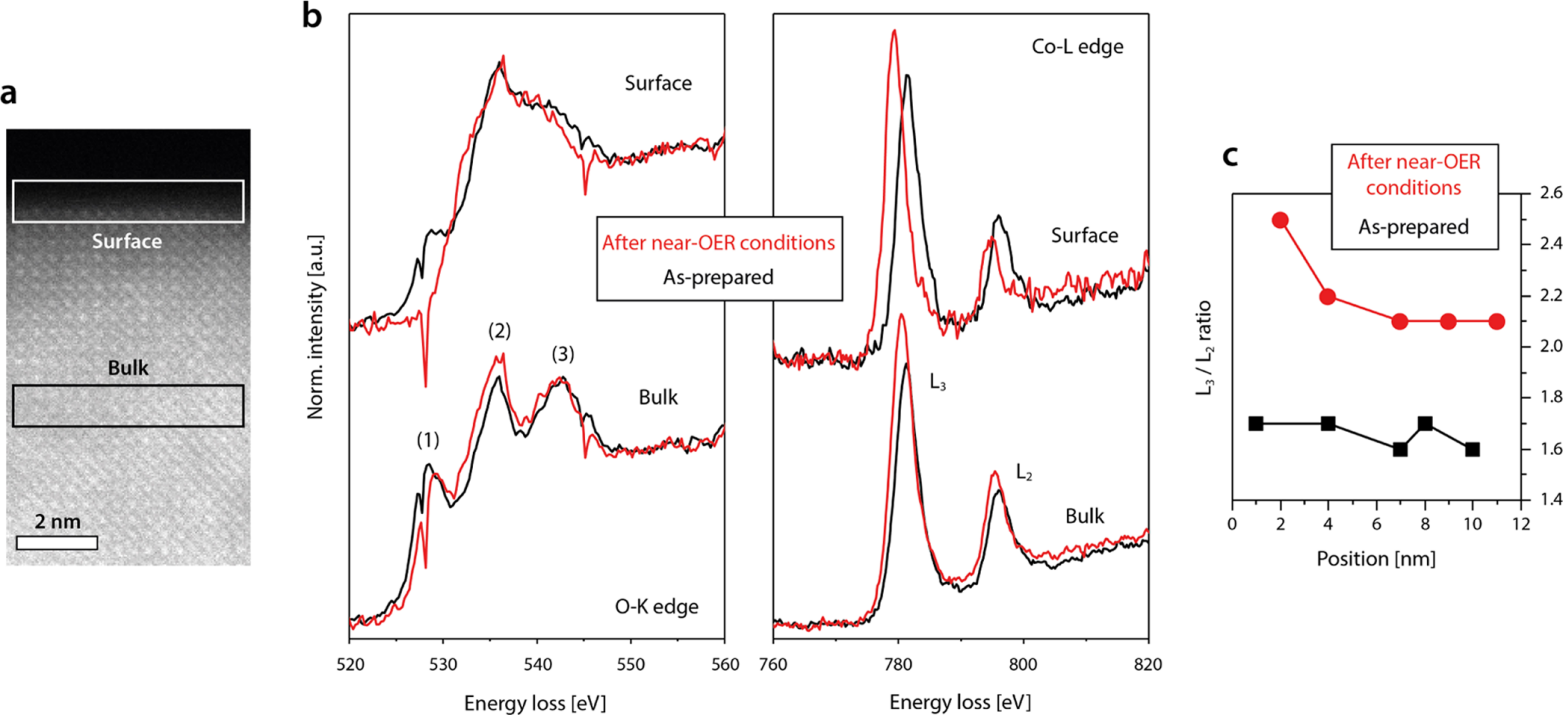
Electron energy-loss spectroscopy. EELS analysis is performed in the as-prepared state (O_2_) and after near-OER conditions after transfer to vacuum (1 × 10^–5^ Pa) of the LSCO catalyst. (a) Measurement position is varied between the surface and the subsurface region of the lamella as highlighted by the white (surface) and black (bulk) rectangles denoted in the ADF-STEM image. (b) O-K-edge and Co-L-edge spectra recorded by EELS. (c) Plot of the Co-L_3_/Co-L_2_ intensity ratio vs the sampling position, where 1 corresponds to the topmost surface. A systematic increase in the Co-L_3_/Co-L_2_ ratio after near-OER conditions indicates a decrease of the average oxidation state of Co toward the catalyst surface. The spectra are background-subtracted and energy-calibrated using the corresponding zero-loss peak.

OER activity is typically directly linked to the local electronic structure at the active catalyst surface. Therefore, electron energy-loss spectroscopy (EELS) is performed to investigate the evolution of the electronic structure of the LSCO catalysts under near-OER conditions. The O-K-edge and Co-L-edge spectra are acquired in the surface region of the catalyst as well as in the bulk (Figure 4a). The formation of a disordered surface layer under near-OER conditions is associated with gradual changes in the electronic signature toward the surface (Figure 4b,c). The O-K-edge exhibits three characteristic peaks denoted as (1), (2), and (3). Here, prepeak (1) is attributed to the hybridization of the O 2*p* states with the Co 3*d* states and indicates the presence of O 2*p* holes, while peaks (2) and (3) represent transitions into hybridized O 2*p*-Co 4*sp* states.^37,38^ The decrease in the height of prepeak (1) obtained in the topmost atomic layers after operation of the sample at near-OER conditions indicates the decrease in the average Co valence state,^39^ which furthermore is in good agreement with the apparent shift in the Co-L-edge toward lower energies.

This observation is consistent with the change in the intensity ratio of the Co-L_3_/Co-L_2_ signals, which is sensitive to the valence state of transition metals, where the average cation valence decreases with increasing Co-L_3_/Co-L_2_ intensity ratio.^37^ As can be seen, the ratio is generally increased after treatment at near-OER conditions, which indicates a reduction of the average oxidation state of Co cations.^40^ Moreover, a distinct increase of the Co-L_3_/Co-L_2_ intensity ratio is visible with decreasing distance to the topmost surface (Figure 4c), which further emphasizes that the electronic changes evident by EELS appear to be interrelated to the surface processes driven under near-OER conditions. In comparison, only a small decrease in the intensity but no change in the spectral shape or shift in the peak position is detected for the La-M-edge spectra in the surface region (Figure S5).

### Evolution of the Surface Chemistry from Perovskite Toward Mixed Phases

**Figure 5:**
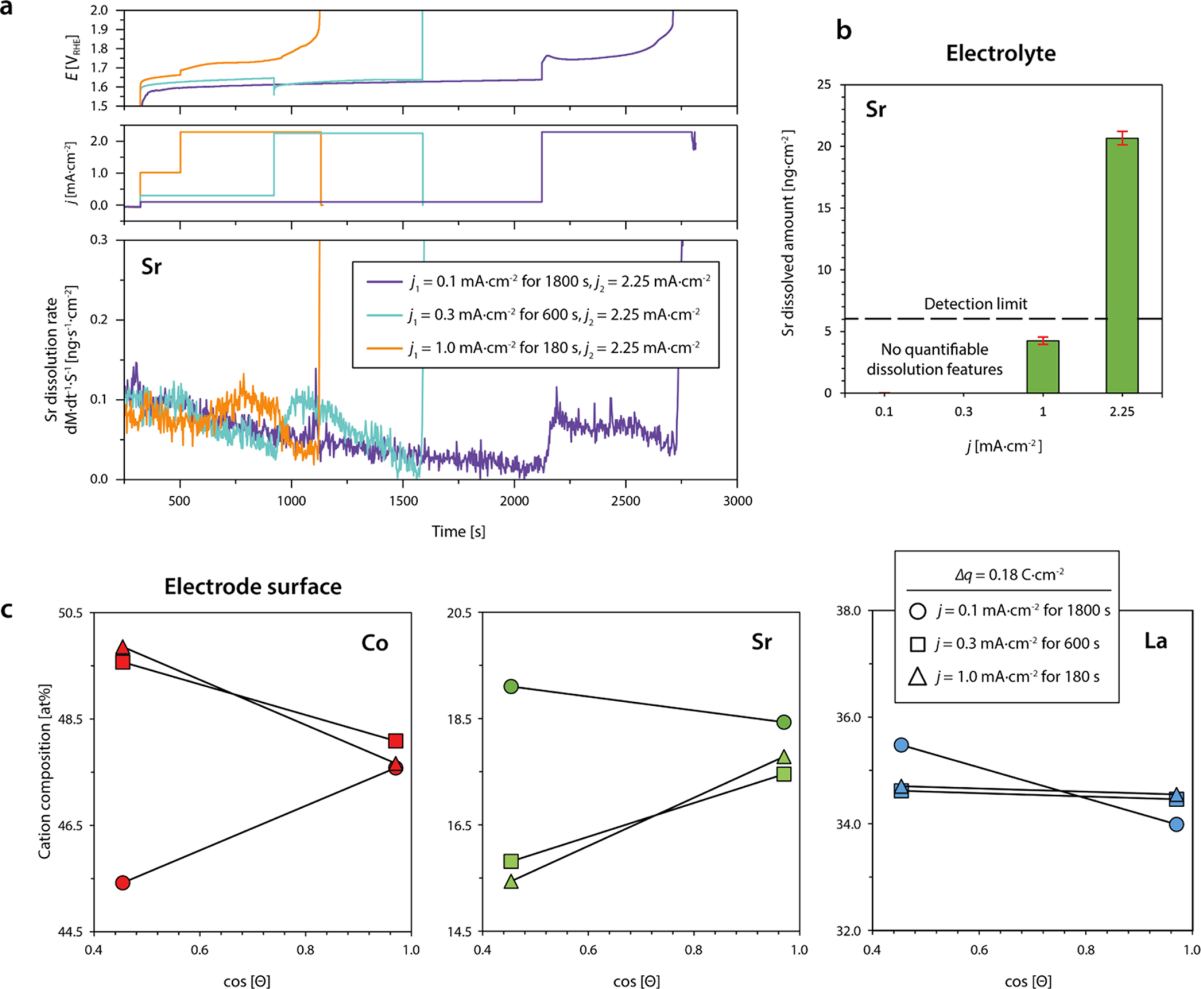
Investigation of stoichiometric changes of LSCO electrocatalysts during steady-state OER operation. (a) Chemical analysis of the electrolyte by online ICP-MS measurements. The dissolution rate of Sr is monitored during three different measurement protocols. For the systematic investigation of Sr dissolution at different current densities, two galvanostatic holds are applied during each measurement protocol, respectively (*j*_1_ = 0.1, 0.3, or 1.0 mA·cm^–2^ and *j*_2_ = 2.25 mA·cm^–2^). (b) Dissolved amounts of Sr are determined for the galvanostatic holds at different current densities. Error bars represent the standard deviation of average values obtained from three measurements, each performed on a fresh catalyst surface. (c) Angle-dependent XPS analysis of the surface stoichiometry after OER operation in the low-potential regime similar to the conditions used during online ICP-MS. The depletion of strontium and the enrichment of cobalt is detected for catalysts operated at *j* = 0.3 and 1.0 mA·cm^–2^. XPS analysis was performed at a photoemission angle Θ = 15° (less surface-sensitive) and Θ = 64° (more surface-sensitive).

To link the structural evolution and electronic changes at the perovskite electrode surface upon OER operation with compositional changes in the near-surface region, a combined approach of online inductively coupled-plasma mass spectrometry (ICP-MS) for the investigation of the leaching behavior and angle-dependent X-ray photoelectron spectroscopy (XPS) analysis for probing the electrode surface region is applied (Figure 5). Online ICP-MS is performed to determine the rate of cation dissolution under electrochemical bias in real time, enabling immediate tracking of dissolved catalyst constituents. The contact between the LSCO thin-film electrodes and the scanning flow cell (SFC) setup was established under 1.0 V vs RHE applied potential, followed by a first galvanostatic hold at different current densities of *j*_1_ = 0.1, 0.3, or 1.0 mA·cm^–2^. The duration of each galvanostatic hold was set to yield the identical charge passed (1800, 600, and 180 s, respectively, Δ*q* = 0.18 C). Each measurement protocol was finished with a second galvanostatic hold at *j*_2_ = 2.25 mA·cm^–2^. This protocol was chosen to test the electrochemical stability under different potentials and to correlate chemical leaching rates with the respective OER reaction rates (via *j*). Throughout the measurements, only the dissolution of strontium was detected with generally low dissolution rates, which indicates a selective leaching of Sr cations from A-sites.

Here, the first significant dissolution feature is typically observed during the potentiostatic hold prior to switching to the galvanostatic holds. This feature is related to the dissolution of minor Sr-rich secondary phases, which are commonly present at the LSCO surface and is visible when the contact between the scanning flow cell is established with the LSCO electrode surface (cf. Figure S6).^41,42^

Figure 5a compares the detected Sr dissolution rate for the three different measurement protocols. Here, a small transient decay in Sr dissolution is observed independent of the applied potential owing to a long tail of the initial contact peak, while distinct dissolution features are only visible for potential steps that result in increased current densities. Interestingly, potential-dependent leaching behavior is evident as summarized in Figure 5b, where Sr dissolution is only detectable during OER operation at a current density *j*_1_ ≥ 1.0 mA·cm^–2^ yielding around 4.3 ng·cm^–2^. Here, the dissolution rate increases in a transient manner and slowly decays until the end of the hold. While qualitatively, a dissolution feature can be observed at *j*_1_ = 1.0 mA·cm^–2^, the calculated dissolved amount of Sr needs to be taken with care at these operation conditions since the value is below the nominal detection limit of the online ICP-MS system.

Galvanostatic holds at a current density of *j*_2_ = 2.25 mA·cm^–2^, however, are accompanied with increased Sr dissolution rates and a total amount of dissolved Sr of about 20.7 ± 0.5, 25.1 ± 5.0, and 21.7 ± 5.8 ng·cm^–2^. Similar to the dissolution feature detected at a lower current density (*j*_1_ = 1.0 mA·cm^–2^), Sr dissolution rapidly increases during the potential step. Each experiment was completed when the contact was lost due to vigorous bubble formation at the working electrode surface, blocking the channels of the SFC. Independent of the specific measurement protocol, the deactivation of the LSCO sample (contact loss) occurred when similar amounts of Sr (∼25 ng·cm^–2^) leached from the crystal lattice for all measurement protocols applied. Our results are consistent with precedent literature data, where the authors observed increased Sr leaching with increasing Sr substitution at the A-site of the perovskite lattice. Remarkably, the authors found that Sr dissolution in LSCO is coupled to the dissolution of small amounts of cobalt.^43^

Again, the nonlinear dependence of the dissolution rate on the applied current density indicates that OER catalysis driven at higher current densities promotes the preferential leaching of A-site strontium cations with a higher rate, consistent with the nonlinear behavior of lifetime and charge (Figure 1e). Based on the online ICP-MS analysis of active LSCO electrocatalysts in combination with blank measurements and the calibration curve recorded for Co, a theoretical S number of 1.89 × 10^6^ can be calculated for cobalt using the galvanostatic holds performed at 2.25 mA·cm^–2^ for 30 min, which can serve as a metric for the catalyst stability (which underestimates the real stability of Co). Remarkably, the theoretical S number for LSCO is in the ballpark of what was calculated for the state-of-the-art crystalline IrO_2_ OER catalyst in acidic media.^20^

To correlate the dissolution behavior with stoichiometric changes at the electrode surface, angle-dependent XPS analysis is performed (Figures 5c and S7). Here, the relative cation composition of the electrode surface is determined after transfer of an equal amount of charge during OER at different current densities of *j* = 0.1, 0.3, and 1.0 mA·cm^–2^. Large photoemission angles Θ allow for the detection of near-surface signals (mean escape depth *d* ∼ 0.6 nm for Co 2*p*_3/2_), while smaller photoemission angles result in the detection of photoelectrons originating from larger information depth (mean escape depth *d* ∼ 1.3 nm for Co 2*p*_3/2_). As can be seen, the surface of the LSCO model electrodes remains predominantly A-site terminated when operated at low current densities of *j* = 0.1 mA·cm^–2^ (cf. Figure S8 for as-prepared state). After operation at higher current densities of *j* = 0.3 and 1.0 mA·cm^–2^ we observe a relative increase of the cobalt signal and decrease of the strontium signal for small Θ, while the lanthanum signal exhibits only minor changes. In contrast, the relative cation composition appears to remain unchanged in the (buried) near-surface region of the perovskite catalyst (large Θ).

Consistent with our online ICP-MS measurements, Sr depletion is promoted at high current densities, which is evidently accompanied by an enrichment of cobalt at the LSCO surface. The XPS investigations reveal, however, that stoichiometric changes at the electrode surface are induced even at low current densities, *j* < 1.0 mA·cm^–2^, while Sr dissolution was detected by online ICP-MS only during operation at *j* ≥ 1.0 mA·cm^–2^. Here, the small dissolution rate of Sr may prevent its detection by time-resolved ICP-MS analysis.

### Identification of Mixed Surface Phases

**Figure 6:**
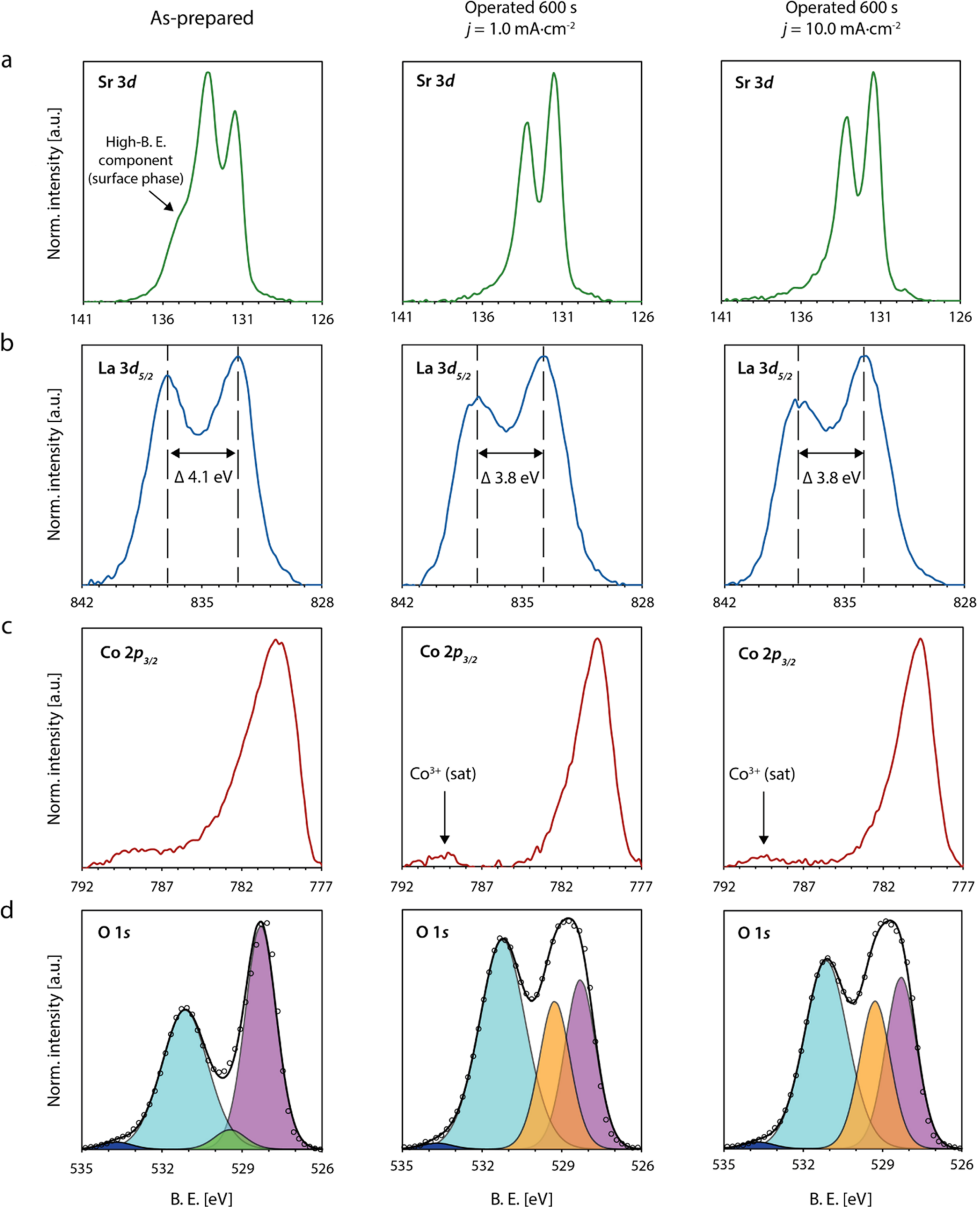
X-ray photoelectron spectroscopy investigations of the LSCO surface chemistry. (a) Sr 3*d*, (b) La 3*d*_5/2_, (c) Co 2*p*_3/2_, and (d) O 1*s* XPS core-level spectra of LSCO catalysts in the as-prepared state (first column), and the operated (but still active) state after OER catalysis for 600 s at *j* = 1.0 mA·cm^–2^ (second column) and the operated (but still active) state after OER catalysis for 600 s at *j* = 10.0 mA·cm^–2^ (third column). The respective spectra are displayed for each sample state after subtraction of a Tougaard background. The O 1*s* spectra are deconvoluted by data fitting of five different components (ABO_3_ lattice oxygen (purple), surface termination component (green), oxyhydroxide lattice oxygen (orange), mixed hydroxide groups (cyan), and organic components (blue)). XPS analysis was performed at a photoemission angle Θ = 46.

To understand the nature of the chemical changes at the LSCO surface during steady-state OER conditions, galvanostatic holds at *j* = 1.0 and 10.0 mA·cm^–2^ are applied and XPS core-level spectra of the as-prepared LSCO surface and the operated catalysts are recorded (Figure 6). Both operated catalysts were still active after OER operation and transferred to the vacuum of the XPS within ∼2 min to limit post-experimental aging.

The Sr 3*d* spectrum of the as-prepared sample, exhibits a shoulder at high binding energies, which is typically related to Sr-rich surface phases frequently observed for LSCO (Figure 6a).^34,41,42,44,45^ After operation of the catalyst, the high binding energy component is vanished, which is consistent with the online ICP-MS investigations presented above. The La 3*d*_5/2_ spectrum exhibits distinct multiplet splitting with a magnitude of ΔB.E. = 4.1 eV for the as-prepared perovskite oxide. In the operated state, the magnitude of the multiplet splitting is decreased to ΔB.E. = 3.8 eV (Figure 6b). Furthermore, a shift in binding energy is evident by comparison with the as-prepared state. Both observations indicate the formation of La(OH)_3_ at the surface upon OER operation.^46^ The observed dissolution of strontium and the formation of lanthanum hydroxide is in accordance with the high solubility of Sr(OH)_2_ and the low solubility of La(OH)_3_ at pH = 13 (0.1 M KOH).^47^ In addition, changes in the chemistry of the transition metal can be observed. The Co 2*p*_3/2_ signature with Doniach–Sunjic lineshape^48^ is composed of an asymmetric peak and a pronounced tail toward higher binding energies, where the tail feature originates from a set of satellite peaks (Figure 6c).

In the operated state, a main peak of decreased width and increased symmetry is visible, indicating a loss in the metallic character of the catalyst surface. Furthermore, a separation between the main peak and the remaining tail structure, now composed of a single satellite peak, becomes apparent. While the assignment of a nominal oxidation state for Co in the as-prepared state of the LSCO catalyst which exhibits metallic character is not physical,^26^ the changes in the relative weight of the satellite features give evidence about the formation of a new surface component at the operated catalysts, which exhibits an oxidation state of Co(III), reflected by the pronounced satellite peak around ∼790 eV.^49,50^

Further insights on the chemical nature of the cobalt surface phase are gained based on the O 1*s* core-level signature (Figure 6d). Deconvolution of the O 1*s* core-level spectrum is based on five different chemical states that can be assigned to the lattice oxygen of the LSCO perovskite oxide, a termination layer component or, after OER operation, a (Co)oxyhydroxide lattice oxygen component, a mixed hydroxide component, and a small amount of organic compounds.

The O 1*s* signature exhibits clear changes in the relative intensity of the different components after electrochemical operation, in particular, visible in the form of a decreased perovskite signal. The changes in the O 1*s* signature are most likely associated with a change in the cobalt chemistry. Most likely, the correlated changes in the spectroscopic signature of the Co 2*p*_3/2_ and the O 1*s* core-level regions indicate the presence of cobalt oxyhydroxide at the catalyst surface. The evolution of a Co(III) signature is accompanied by the emergence of an additional peak contributing to the O 1*s* signal, which is in accordance with the two different chemical states of oxygen (single bond and double bond) within the CoO(OH) compound. While the signal of the CoO(OH) hydroxide groups contributes to the mixed hydroxide component, the newly evolved component (orange peak in Figure 6), visible only for the operated state, likely reflects the contribution of the double bonded oxygen in CoO(OH).^50^ The presence of a lanthanum oxyhydroxide compound can be excluded due to its inherent instability in aqueous solution.^51^ Although indications for changes in the Co oxidation state were detected in previous operando ambient pressure XPS studies of La_0.8_Sr_0.2_CoO_3-δ_, the evolution of a pronounced Co-oxyhydroxide signature in the O 1*s* core-level spectrum has not been observed.^52^ Here, the lower strontium content, which is typically associated with a lower catalytic activity within the La_1–*x*_Sr*_x_*CoO_3−δ_ group may result in a higher stability and slower transformation of the perovskite surface. Interestingly, significant changes in the cobalt oxidation state were observed by operando XAS studies of La_0.6_Sr_0.4_CoO_3−δ_ in the near-surface region, while for an LaCoO_3−δ_ reference sample, little to no changes in the cobalt valence were detected.^53^ Here, the authors report the reduction in the Co oxidation state at open-circuit potential, while an oxidation of cobalt is observed under an applied potential of 1.4 V vs RHE, where the authors assume an intact perovskite surface of the LSCO catalyst at these rather mild conditions. Both studies hence emphasize the important role of Sr doping for the evolution in the surface chemistry of LSCO electrocatalysts under OER conditions, which is furthermore related to the transient degradation behavior described in the present study.

The phase transition of the perovskite surface of mixed cobalt valency of nominally (III)/(IV) character toward a Co-oxyhydroxide phase with an oxidation state of Co(III) is consistent with the EELS results presented above, which have indicated a reduction of the average Co oxidation state of the operated catalyst surface. Furthermore, the observation of CoO(OH) formation is consistent with recent reports for LSCO OER electrocatalysts.^54^ Interestingly, the cobalt oxyhydroxide component was vanished after the end of lifetime, leaving behind a Co 2*p*_3/2_ and O 1*s* signature similar to the initial state, while a clear La(OH)_3_ signature remains visible (cf. Figure S9). However, it cannot be ruled out that these post-mortem changes result from the rapidly increasing potential at the end of the catalyst lifetime in galvanostatic measurements.

Our findings emphasize the particular importance of dynamic surface transformations at the surface of the perovskite OER catalyst. These processes are accompanied with severe changes of the catalyst surface properties, which occur within the initial phase of OER catalysis at the solid–liquid interface.

## Discussion

In summary, we demonstrate that under OER conditions, an active state of the LSCO catalyst surface rapidly evolves where highly mobile Co species dominate the surface chemistry, while additional irreversible processes result in an altered surface chemistry associated with a surface-dominated degradation process on longer time scales. The clear interrelation between the applied potential and the chemical changes of the perovskite surface may support the hypothesis that the process is driven by the potential-induced lattice oxygen evolution reaction.^55,56^ Here, the evolution of molecular oxygen, which originates from oxygen anions of the perovskite lattice, results in the decomposition of the perovskite structure and the release of Sr, La, and Co cations.

**Figure 7:**
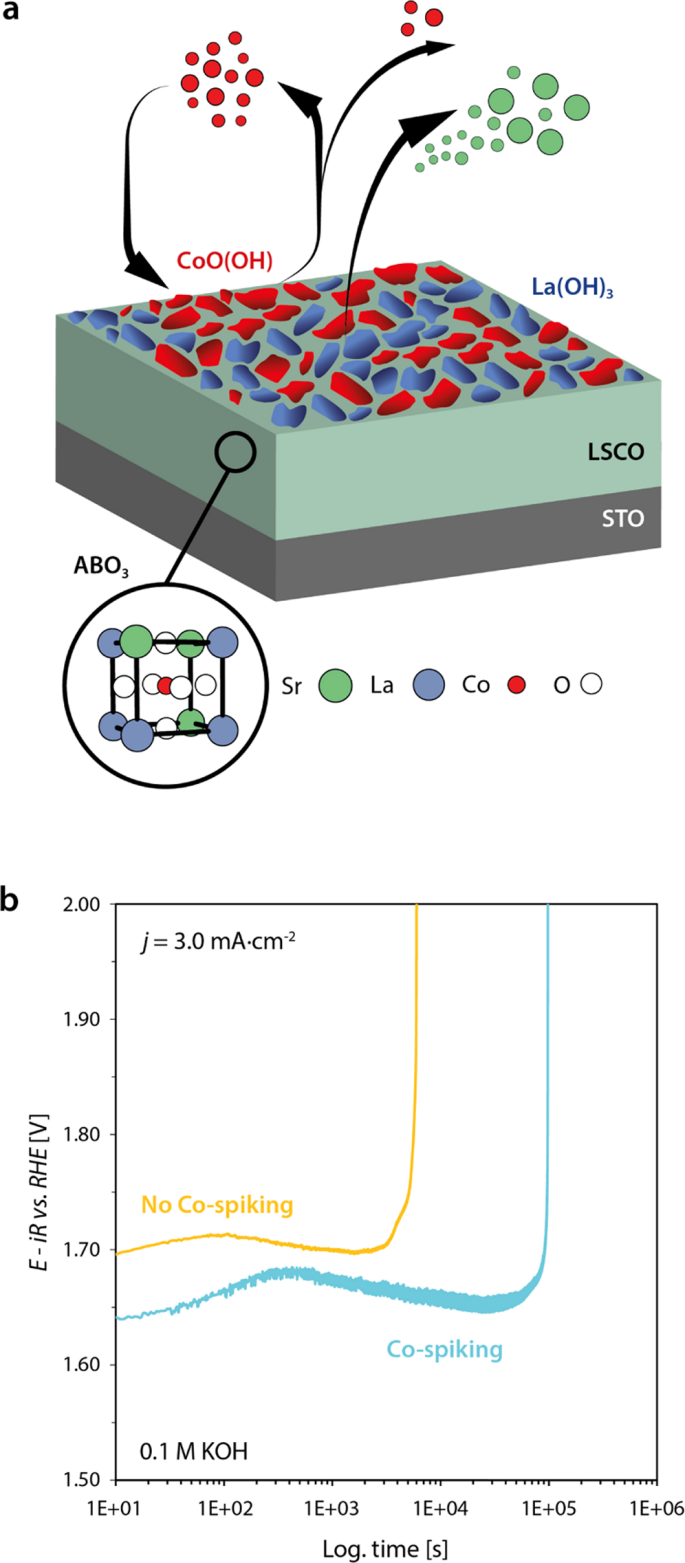
(a) Schematic illustration of the dynamic surface transformations induced under OER conditions. Strontium leaching and Co hopping during the initial phase of OER result in the formation of mixed phases at the catalyst surface. A dynamically evolving and catalytically active CoO(OH) phase as well as a highly insoluble and catalytically inactive La(OH)_3_ phase is formed. (b) Chronopotentiometric measurements for the characterization of the LSCO stability on the basis of the catalyst lifetimes at an applied current density of *j* = 3.0 mA·cm^–2^ comparing the lifetime of LSCO in 0.1 M KOH and Co-spiked 0.1 M KOH. Abrupt increase of potential denotes the end of lifetime. The catalyst lifetime is considerably increased by Co-spiking of the electrolyte.

The subsequent stoichiometric evolution of the catalyst surface under applied potential appears to be mostly determined by the solubility of the involved cations in the investigated potential-pH window,^47^ resulting in a complex evolution of mixed oxide phases at the catalyst surface. The catalyst surface becomes depleted from highly soluble strontium cations, while cobalt of lower solubility is enriched at the surface under initial OER operation, as illustrated in Figure 7a. In contrast, lanthanum is insoluble under the given conditions and consistently, lanthanum stoichiometry appears to be widely unchanged across the near-surface region. The topmost surface of operated LSCO electrocatalysts is found to be composed of La(OH)_3_ and CoO(OH) (Figure 7a). While Co-oxyhydroxide was reported to actively catalyze the OER,^8^ lanthanum hydroxide may play a particular role in the deactivation mechanism of LSCO electrocatalysts, since it does not participate in oxidation reactions^57^ and is highly stable under OER conditions.^47^ Therefore, the compound may have a passivating character and likely blocks parts of the active catalyst surface. After the assembly of an active Co-oxyhydroxide layer as well as an inert lanthanum-hydroxide phase under applied potential, OER catalysis will be widely determined by the equilibrium between the dynamic dissolution of CoO(OH) and its redeposition.^8^

Considering the dynamic processes at the catalyst surface, we propose that the dynamic equilibrium successively shifts toward Co dissolution with increasing potentials, which results in the gradual loss of cobalt from the active oxyhydroxide layer to the electrolyte over the course of many completed oxygen evolution reaction cycles. Thus, the depletion of Co-oxyhydroxide from the self-assembled surface layer is accelerated at high OER rates, which may result in a failure of the catalyst when a critical ratio between CoO(OH)/La(OH)_3_ coverage of the surface is reached. In addition, agglomeration of the CoO(OH) surface phase may result in a decrease of the catalytically active surface area. As shown in Figure 7b, this process can be delayed by spiking the electrolyte by cobalt, following the report of Chung et al.^58^ In this way, the balance between Co-oxyhydroxide and La-hydroxide can be manipulated by shifting the equilibrium between Co dissolution and redeposition toward the precipitation of the solid Co-oxyhydroxide phase, which yields a 16-fold increase in lifetime.

Our findings suggest that not only the activity of the LSCO catalysts is determined by the assembly of active surface phases but also that the overall lifetime of the electrocatalysts is mainly determined by the stability of the active Co-oxyhydroxide surface layer. Here, it is crucial to maintain the balance between dissolution and redeposition of Co, strongly influenced by the operation conditions, to preserve the active Co-oxyhydroxide phase. Potential-dependent surface processes hence may result in the successive degradation of the catalytically active surface layer under steady-state operation conditions, which may inherently limit the lifetime of perovskite electrocatalysts at increased, technically relevant current densities.

## Conclusions

Under steady-state OER operation, an active surface state of LSCO electrocatalysts rapidly evolves, which is characterized by Co-oxyhydroxide species that coincides with high ion dynamics at the solid–liquid interface. On longer time scales, degradation of the LSCO electrocatalysts is observed. While the catalyst bulk remains mostly unaffected at constant load, the catalyst lifetime is critically limited by the dynamic transformations of the topmost perovskite surface toward mixed phases. The degradation behavior hence considerably differs from dynamic conditions, where excessive amorphization and dissolution processes typically result in a loss of the catalytic performance over time. The surface transformation is potential-induced and results in a limited efficacy of the LSCO electrocatalysts in the high potential regime. We show that dynamic hopping of Co species across the LSCO surface is facilitated in the presence of an aqueous electrolyte, i.e., at the solid–liquid interface, which most likely plays an important role in the dynamic structural and chemical evolution of the catalyst surface. While Sr readily dissolves in the electrolyte at relevant OER potentials, La remains stable at the surface in the form of La(OH)_3_ and Co enriches at the surface in the form of CoO(OH) during the early stage of steady-state OER operation. We propose that the interplay of the catalytically active and dynamically evolving CoO(OH) and a highly stable and catalytically inactive La(OH)_3_ surface phase results in potential-induced deactivation of the catalyst. Here, the gradual dissolution of Co in the electrolyte, i.e., successive passivation of the LSCO surface by La(OH)_3_, causes the catalyst failure. The dynamic transformations in the surface chemistry that are driven under OER conditions are therefore key to understand not only activity trends but also stability limitations of perovskite electrocatalysts.

## Experimental Section

### Thin-Film Fabrication

Epitaxial La_0.6_Sr_0.4_CoO_3-δ_ thin-film model electrodes of 20 nm thickness were deposited on single-crystalline, epi-polished (001) SrTiO_3_ (STO) and (001) Nb(0.5 wt %):SrTiO_3_ (Nb:STO) substrates (Shinkosha Co. Ltd., Yokohama, Japan) in [001] orientation. The sample size is 0.5 × 10 × 10 mm^3^. The deposition process was performed by reflection high-energy electron-diffraction-controlled pulsed laser deposition at an oxygen partial pressure of *p*(O_2_) = 0.053 mbar. The growth temperature was *T* = 650 °C and the laser fluence *F* = 2.19 J·cm^–2^ using a repetition rate of *f* = 5 Hz. The distance between the ceramic target and the heated substrate was *d* = 60 mm and a nanosecond KrF-excimer laser with a wavelength of λ = 248 nm was used to operate the PLD system. Platinum electrodes with a thickness of 50 nm were sputtered at the edges of the oxide thin-film surface to provide sufficient contact with the potentiostat. Furthermore, the backside and the sides of the substrates were covered with 50 nm of Pt forming a contact with the front Pt pads. Conductive Nb:STO substrates were applied to provide an additional pathway for charge transfer through the Nb:STO/LSCO interface to improve measurement geometry for individual probing techniques when required. Please note that the use of undoped and Nb-doped STO substrates may result in slight differences in the potential drop within the sample and hence was only applied when an influence on the consistency of data interpretation can be excluded.

### Thin-Film Characterization

The surface morphology of the thin-film electrodes was investigated using atomic force microscopy (AFM, Cypher, Oxford Instruments Asylum Research Inc., Santa Barbara) operated with a tip with a curvature of ∼8 nm. The crystal structure of the thin films was characterized by X-ray diffraction (XRD, D8 Discover, Bruker AXS GmbH, Karlsruhe, Germany) by symmetric 2θ–ω scans around the (002) reflections as well as reciprocal space mapping (RSM) using asymmetric scans around the (013) reflections in grazing exit geometry. The diffractometer was equipped with a goebel mirror, a Cu K_α_ monochromator, a centric Eulerian cradle and a Lynxeye XE detector. To provide for lateral resolution, a pinhole adapter of 2 mm diameter was applied. X-ray photoelectron spectroscopy (XPS, Phi 5000 VersaProbe, ULVAC Phi, Physical Electronics, Inc.) was applied to study the surface chemistry using the Al K_α_1__ line (*E*_λ_ = 1486.6 eV, FWHM = 0.26 eV) of a monochromized X-ray source and constant pass energy (*E*_0_ = 29.35 eV) in fixed analyzer transmission mode. To vary between different information depths of the detected photoelectrons, photoemission angles of Θ = 15 and 64° (cf. Figures 5c, S7, and S8) were applied for the XPS analysis, while the XPS spectra presented in Figures 6 and S9 were recorded at Θ = 46°. To calculate the cation stoichiometry, relative sensitivity factors (RSF) were referenced to the ceramic target material. For peak fitting, the full width at half-maximum (FWHM) of the components was constrained to exhibit an equal value and the components have a fixed position on the B.E. scale for comparison of different samples. However, the mixed hydroxide component is based on the sum of various overlapping signals of hydroxide groups with slightly varying binding energy due to differences in the chemical environment. Consequently, broadening of the multicomponent peak compared to the other involved (single-component) chemical states requires a larger FWHM applied for peak fitting. For data evaluation, a Tougaard-type background was subtracted and the binding energies of all spectra were aligned to the C 1*s* signal. The inelastic mean free path (1lambda IMFP) was calculated by the QUASES-IMFP software using the TPP2M formula^59^ on the basis of the material properties of as-prepared LSCO. The mean escape depth was determined as an indicator for the angle-dependent information depth of the XPS core level.^60^ The energy scale was periodically calibrated to the Au 4*f* core-level spectrum of a reference sample. XPS analysis of the operated samples was performed after rapid transfer (*t* ∼2 min) to UHV subsequent to electrochemical operation and after gently patting the sample dry with a clean room swipe under N_2_ atmosphere.

### TEM Sample Preparation

Three ultrathin TEM lamellae were prepared from epitaxial (LSCO) (001) thin films with a thickness of 100 nm deposited on a single-crystalline NdGaO_3_ (NGO) substrate in orthorhombic (110) surface orientation. The lamellae were prepared using an Alkali Resistant Positive Photoresist X AR-P 5900/4 protection layer by means of focused ion beam (FIB) on a DENS solutions heating and basing chip for in situ TEM measurements. Here, each lamella is attached to one of the electrical contact of the chip with +20° offset. Platinum (1 μm thickness) is deposited to establish an electrical contact with the thin film to measure the beam-induced potential. Here, Pt is deposited far from the region of interest, so there is no possibility of interference of Pt in the in situ ETEM catalytic investigations. Figure S1a shows the region of interest for the in situ ETEM observations. Primary electron beam-induced secondary electron emission results in a potential of 1.5 ± 0.2 V, which is close to relevant OER potentials. To remove a minor amorphous layer that forms on the surface upon FIB preparation, an electron beam-induced surface recrystallization procedure is performed in situ under 1 mbar oxygen partial pressure at *T* = 400 °C. Additional lamellae were prepared on a Cu grid for EELS analysis.

### Environmental Transmission Electron Microscopy

To evaluate the processes at the catalyst surface over time, several ETEM movies are recorded with a small negative defocus that results in a dark contrast of all atomic columns. In addition, a through focus series was acquired in O_2_ gas for contrast simulation. In situ ETEM experiments are carried out using a FEI Titan ETEM G2 80-300 at an operating voltage of 300 kV, equipped with a Cs corrector. All in situ movies are recorded using a Gatan UltraScan 1000XP at a beam current of 4 nA. The movie in O_2_ is recorded with a cold trap to decrease the H_2_O partial pressure. Local electron dose rates at the location of TEM lamella surfaces are measured by calibrated CCD contrast with 0.16696 electrons/count, yielding 19.955 and 35.654 e/Å^–2^ s^–1^ for O_2_ and H_2_O environments, respectively. A careful analysis of beam effects excludes that an observed increase in Co mobility is induced by the momentum transfer during scattering of the high energetic primary electrons and give strong evidence for thermally induced surface hopping that is enhanced by H_2_O. The electron beam-induced potential was measured via the electronic contact to the LSCO film and the DENS solutions holder as well as a FIB-Pt bridge across the substrate to be 1.5(±0.2) V with respect to the ground (i.e., TEM column). An impedance converter was used to maintain nearly open-circuit conditions during the voltage measurement.

### Electron Energy-Loss Spectroscopy

The EELS analysis are performed using a Gatan Quantum 965ER post-column energy filter in the ETEM. Spectra of Co-L-, O-K-, and L-M-edges are acquired using 0.25 eV/ch dispersion in 1 mbar O_2_ and post 5 μbar H_2_O. Power-law background functions from Gatan’s Digital Micrograph are fitted to a 50 eV wide window before each Co-L-edge, 25 eV for O-K-edge, and 23 eV for La-M-edge for background subtraction. Python-based code is used for the precise calculation of the L_3_/L_2_ ratio. The spectra are background-subtracted and energy-calibrated using the corresponding zero-loss peak.

### Image Simulation

Multislice simulations of HRTEM images are conducted with QSTEM^61^ following the procedure described in ref (35). For this purpose, the sample thickness as well as relevant electron-optical parameters are determined by minimizing the root-mean-square difference between experimental and simulated images of a single unit cell using the Metropolis algorithm. Multislice image simulations were performed using a sample thickness of 1.53 nm, a defocus of −14.5 nm, and a focal spread of 9 nm. Twofold astigmatism as well as the spherical aberration are found to have only a small influence on the image contrast within their experimental uncertainty and thus set to zero to avoid overfitting. In a second step, the obtained parameters are used during the simulation of a larger supercell including an A-site-terminated surface.

### Electrochemical Characterization

Electrochemical characterization of the thin-film electrodes was performed in a rotating disc electrode (RDE) setup (Pine Research) in O_2_-saturated 0.1 M KOH with a rotation rate of 1600 rpm using a custom-made adapter for 0.5 × 10 × 10 mm^3^-sized thin-film samples. A chemically resistant Teflon beaker was applied as the electrochemical cell and O_2_-saturated (continuous purging) 0.1 M KOH was applied as the electrolyte, which was prepared by dissolution of KOH pellets (Sigma-Aldrich, 99.99%) in deionized water (Milli-Q, >18.2 MΩ cm). The potentiostat (BioLogic SP-150, Bio-Logic Science Instruments, France) was connected to the platinum contact sputtered at the backside of the substrate via a Pt stamp, providing for facile charge transfer to the thin-film working electrode while using a Pt-coil counter electrode. The center of the perovskite catalyst was sealed from the platinum contacts by an o ring with a diameter of *d* = 7.5 mm. The measurements were performed in reference to an Hg/HgO electrode (CHI Instruments), which was experimentally calibrated to the RHE (HydroFlex) for each batch of electrolyte. Electrochemical testing was conducted by electrochemical impedance spectroscopy, scan-rate-dependent cyclic voltammetry in the pseudocapacitive redox phase change region, and cyclic voltammetry in the OER potential region (two cycles successively). For Tafel analysis, chronopotentiometric (staircase) measurements were performed at different potentials in the range of *j* = 0.1–0.8 mA·cm^–2^ with galvanostatic holds of 10 min. For lifetime testing, LSCO thin-film electrodes were operated in the OER region using chronopotentiometry at different current densities between *j* = 0.1 and 10.0 mA·cm^–2^. The entire setup was stored in a glovebox, which was continuously purged with nitrogen gas. All potentials were *iR*-corrected by the uncompensated series resistance *R*_S_ of the electrode setup, that is typically in the range between *R*_S_ = 75 and 100 Ω, as determined using the high-frequency intersect of the Nyquist plot determined by electrochemical impedance spectroscopy.

### Online ICP-MS Measurements

To investigate the stability of the LSCO thin-film samples, the outlet of a custom-designed and manufactured polycarbonate scanning flow cell (SFC) was coupled to the inlet of an inductively coupled-plasma mass spectrometer (ICP-MS, PerkinElmer NexION 350X). The micro flow cell setup allows to probe the electrochemical performance and dissolution behavior at several locations of the same thin-film sample. A glassy carbon rod (SIGRADUR) was used as the counter electrode and an Ag/AgCl/3 M KCl (Metrohm) as the reference electrode. The counter electrode was channeled in the SFC from the inlet side via a T-connector, while the reference electrode was connected through a capillary channel from the outlet side (to avoid Cl^–^ contamination). The LSCO thin films served as the working electrode (measured working electrode surface was 7.85 × 10^–3^ cm^2^). All electrochemical protocols during online stability measurements were performed using a potentiostat (Gamry, Reference 600). The working electrode was placed on an XYZ translation stage (Physik Instrumente M-403), allowing the rapid screening of multiple spots along the same LSCO sample. Electrochemical protocols were performed in 0.05 M KOH electrolyte solution (salt/organic matter content should be less than 2 w/w% for ICP-MS) saturated with Ar. Three different protocols were carried out as follows: contact with the working electrode was established at 1 V vs RHE and the electrode was held at this potential for 5 min. This was followed by either a galvanostatic hold at *j* = 0.1 mA·cm^–2^ for *t* = 30 min or *j* = 0.3 mA·cm^–2^ for *t* = 10 min or *j* = 1 mA·cm^–2^ for *t* = 3 min. All measurements were completed with a galvanostatic hold at *j* = 2.25 mA·cm^–2^ for *t* = 30 min. An *iR* correction of 1 kΩ was applied during all measurements. The total Sr loss during OER operation is quantified by integration of the dissolution features detected by ICP-MS during the respective galvanostatic holds. The ICP-MS was calibrated daily by a four-point calibration slope made from standard solutions (Merck Certipur, Sr, La, In, Nb, Y, Ti, Sc, Co, Ge 1000 mg·L^–1^) containing the metals of interest in a given concentration in 0.05 M KOH. ^115^In (for ^88^Sr, ^139^La), ^89^Y (for ^93^Nb), ^45^Sc (for ^47^Ti), and ^74^Ge (for ^59^Co) served as internal standards. Internal standard solutions were prepared in 1% HNO_3_.The electrolyte flow-rate was controlled by the peristaltic pump of the ICP-MS; the average flow-rate was 3.46 ± 0.03 μL·s^–1^.

## Supporting Information Available

Recrystallization procedure of the FIB lamella, ETEM investigation in O_2_ environment, investigation of the LSCO surface dynamics in 0.5 Pa of H_2_O, EELS analysis of the La-M-edge, online ICP-MS analysis including contact peak, angle-dependent XPS analysis of as-prepared LSCO, and XPS analysis of the surface chemistry of LSCO after the end of the catalyst lifetime (PDF)
Movies M1–M4 (ZIP)

## Abbreviations

AFM: atomic force microscopy
CCD: charge-coupled device
EELS: electron energy-loss spectroscopy
EIS: electrochemical impedance spectroscopy
ETEM: environmental transmission electron microscopy
FIB: focused ion beam
ICP-MS: Inductively coupled-plasma mass spectrometry
LSCO: La_0.6_Sr_0.4_CoO_3−δ_
OER: oxygen evolution reaction
RSM: reciprocal space mapping
SFC: scanning flow cell
XRD: X-ray diffraction
XPS: X-ray photoelectron spectroscopy
