# Supplementary material for: Atomistic Insights into Activation and Degradation of La0.6Sr0.4CoO3−δ Electrocatalysts under Oxygen Evolution Conditions
Source: J Am Chem Soc. 2022 Sep 21;144(39):17966–79. doi: 10.1021/jacs.2c07226 (PMC9545157; doi:10.1021/jacs.2c07226)
Supplement: Supplementary file 1 — ja2c07226_si_001.pdf [file ja2c07226_si_001.pdf]

SUPPORTING INFORMATION

Atomistic insights into activation and degradation of $La_{0.6}Sr_{0.4}CoO_{3-\delta}$ electrocatalysts under oxygen evolution conditions

Moritz L. Weber[a,*], Gaurav Lole[b,c], Attila Kormanyos[d], Alexander Schwiers[a], Lisa Heymann[a], Florian D. Speck[d,e], Tobias Meyer[b], Regina Dittmann[a], Serhiy Cherevko[d], Christian Jooss[b,c], Christoph Baeumer[a,f] and Felix Gunkel[a,*]

[a]Peter Grünberg Institute (PGI-7) and JARA-FIT, Forschungszentrum Jülich GmbH, Jülich, 52425, Germany; Institute of Materials Physics, [b]University of Göttingen, Göttingen, 37077, Germany; [c]International Center for Advanced Studies of Energy Conversion (ICASEC), University of Göttingen, Göttingen, 37077, Germany; [d]Helmholtz-Institute Erlangen-Nürnberg for Renewable Energy (IEK-11), Forschungszentrum Jülich GmbH, Erlangen, 91058, Germany; [e]Department of Chemical and Biological Engineering, Friedrich-Alexander-Universität Erlangen-Nürnberg, Erlangen, 91058, Germany; [f]MESA+ Institute for Nanotechnology, University of Twente, Faculty of Science and Technology, Enschede, 7500 AE, The Netherlands

*Email: mo.weber@fz-juelich.de; f.gunkel@fz-juelich.de

Figure S1. Recrystallization of the FIB lamella for ETEM investigations.

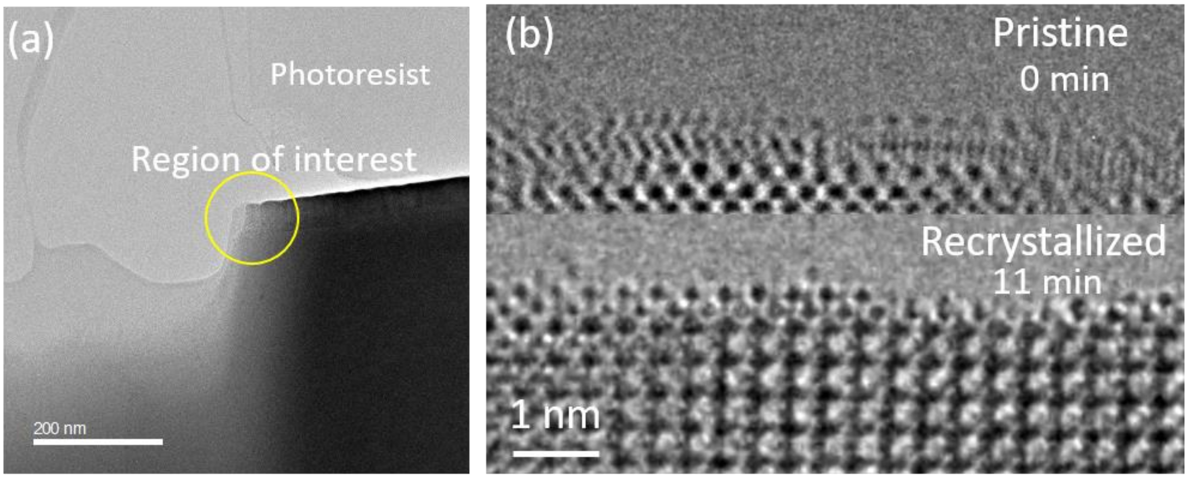

Figure S1. Lamella and surface preparation. (a) TEM image of region of interest (ROI). The photoresist protection layer is removed during lamella thinning procedure in FIB. (b) HRTEM images of the pristine and recrystallized LSCO surface. The thin amorphous layer at the top of LSCO surface formed during ion milling procedure in FIB can be recrystallized by electron irradiation of the surface. The image shows the result after 11 min in 100 Pa of O_2 (dose rate – 10320 e/A^{2}s).

To reduce beam damage during FIB preparation of the LSCO lamella for ETEM investigations, a photoresist protection layer is applied. After removal of the protection layer by FIB thinning, only minor amorphization of the topmost surface is evident (Fig. S1, above). To reconstruct the perovskite structure in order to provide for an atomically sharp interface region, the LSCO surface recrystallized by electron irradiation (Fig. S1, below). For detailed information about the recrystallization procedure, we refer to reference (1).

Figure S2. Observation of a well-ordered and stable surface in O_2.

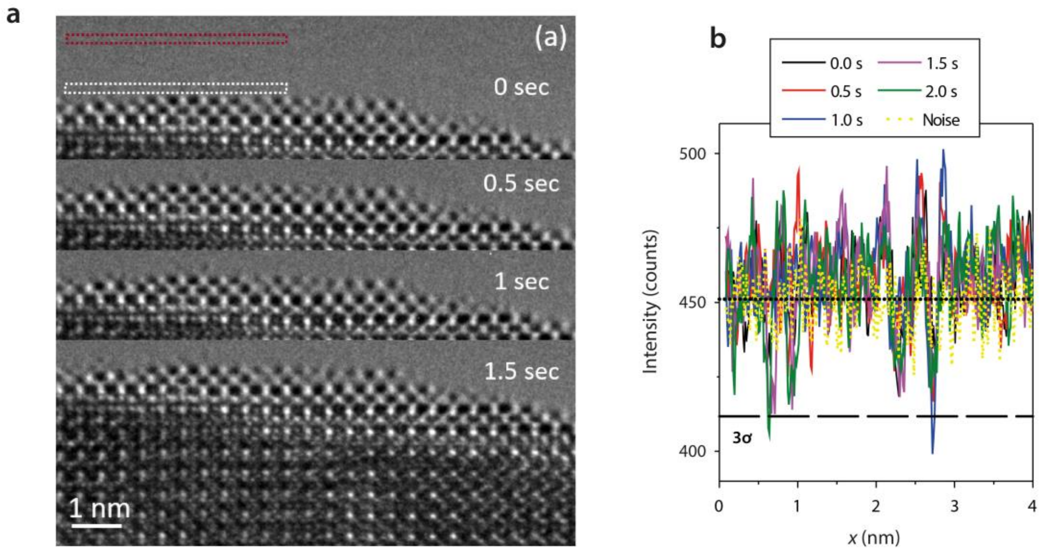

Figure S2. (a) Time sequence of HRTEM images of a (001) LSCO surface from 0 s – 1.5 s recorded in a 2.7 Pa O_2 atmosphere with a frame rate of 2 fps (Movie M1). The surface shows an ordered A-site termination. (b) Line profiles are recorded close above the A-site terminated surface as indicated by the white rectangle in the first image of the time series to quantitatively evaluate the hopping events under oxygen environment. The noise level is determined above (red rectangle) and the 3σ threshold is calculated from the noise signal, which indicates the detection limit of Co adatoms.

After recrystallization of the LSCO surface, a sharp A-site terminated surface is visible, which remains stable in O_2 environment. Notably, the thin film deposition is carried out under oxidizing conditions and hence may result in a similar surface termination. A time series shown in Fig. S2 reveals no considerable contrast fluctuations associated to Co hopping events taking place at the surface under O_2 exposure. The statistically significant detection of transition metal atoms requires triple occupation, i.e. three adatoms lined up in the direction of the incident electron beam on the A-terminated surface. Hence, intensity fluctuations above 3σ reflect the appearance and disappearance of adatom contrast, correlated to the hopping processes of cobalt moieties from the subsurface region onto the surface and vice versa. Only few signals above the 3σ level are evident by line profiles acquired at the interface (white rectangle). The noise level i.e. the background fluctuations of the CCD signal is recorded in the vacuum region above the surface (red rectangle). Given the required triple occupation for the statistically significant detection of Co adatoms and considering the limited frame rate of the CCD detector, a lower limit of the surface adatom hopping rate of 0.5 s^{-1} can be determined for O_2 conditions. In comparison, the lower limit of the surface adatom hopping rate during H_2O exposure was found to be 4.0 s^{-1}, which emphasizes the high mobility of Co species at the LSCO-H_2O interface, under near-OER conditions of the perovskite electrocatalyst.

Figure S3. Redox peak analysis of LSCO by sweep rate-dependent cyclic voltammetry**.**

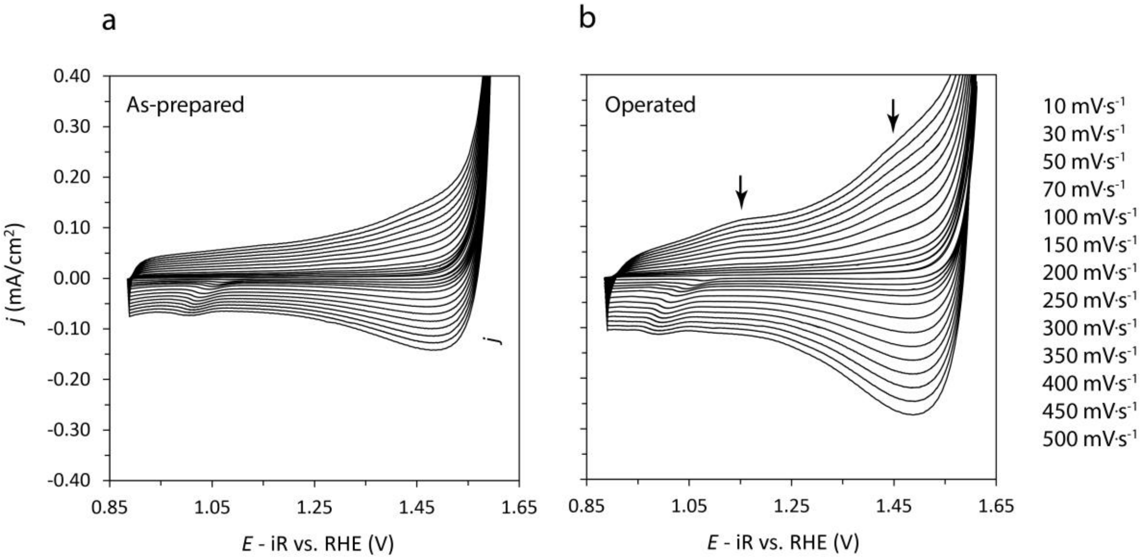

Figure S3. Redox peak analysis of LSCO by sweep rate-dependent cyclic voltammetry in the (a) as-prepared state and (b) in the operated state after ten sweeps to a maximal potential of E = 1.7 V vs. RHE. Broad redox features for the $Co^{2+/3+}$ and $Co^{3+/4+}$ redox couples are highlighted by arrows.

The presence of faint redox features can only be surmised for the as-prepared state of the catalyst (Fig. S3a). In comparison, the magnitude of the exchange current is increased for an operated, but still active catalysts relative to the as-prepared state (Fig. S3b). The redox features are more pronounced after CV cycling, which indicates a higher redox activity of the Co transition metal species after operation of LSCO electrocatalysts, which is consistent with the formation of an active electrode surface under OER conditions.

Figure S4. Investigation of the LSCO surface dynamics in 0.5 Pa of H_2O.

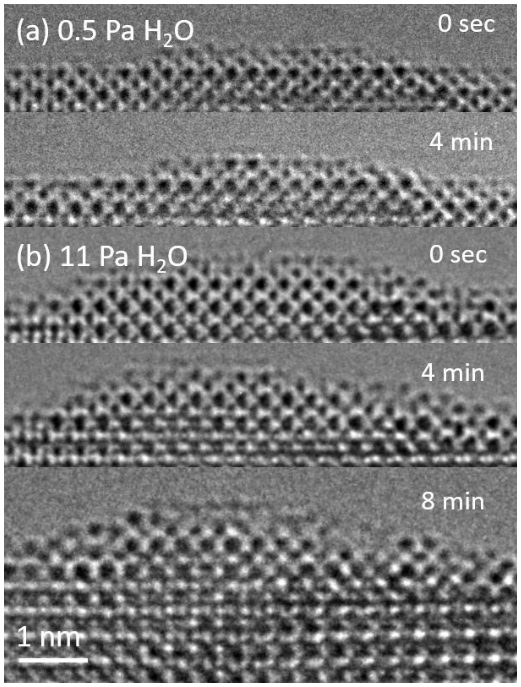

Figure S4. (a) HRTEM frames at 0 sec and 4 min recorded in 0.5 Pa of H_2O showing very slow formation of disordered layer on the surface in low H_2O pressure. (b) HRTEM frames with the time interval in 11 Pa of H_2O taken from the movie M4. Fast formation of a highly dynamical disordered surface in high partial pressure of H_2O.

Fig. S4a shows a series of HRTEM images acquired at the LSCO-H_2O interface over the time of several minutes at equal conditions to Fig. 3c of the main manuscript (0.5 Pa H_2O). Here, the formation of a disordered surface layer proceeds slow in comparison to investigations at increased partial pressures of 11 Pa H_2O (Fig. S4b), where the disordered surface layer forms during a comparably short time imaging time.

Figure S5. EELS analysis of the La-M-edge.

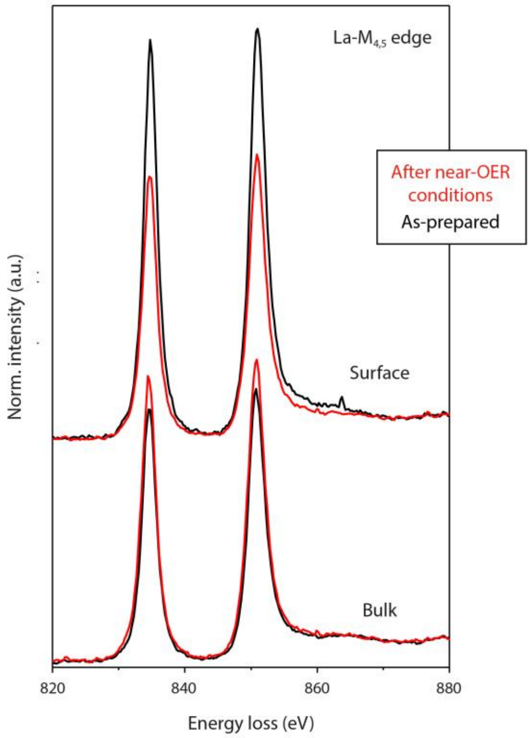

Figure S5. Electron energy loss spectroscopy. EELS analysis of the La-M-edge spectra is performed in the as-prepare state (O_2) and after near-OER conditions after transfer to vacuum (1 x 10^{-5} Pa) of the LSCO catalyst comparing the surface and the bulk region.

Fig. S5 shows the La-M-edge recorded in the as-prepared state and after near-OER conditions and comparing the bulk and surface region of the catalyst. In comparison to the O-K-edge and Co-L-edge only a small differences are visible for the different ample states. Here, a decrease in the intensity but no change in the spectral shape or shift in the peak position is visible for the surface region. No significant shift observed for La M-edge confirms high precision of the energy calibration by zero loss peak and the observed shift in Co L-edge in Fig. 4 of the main text is due to the interaction with H_2O.

Figure S6. On-line ICP-MS analysis of strontium dissolution.

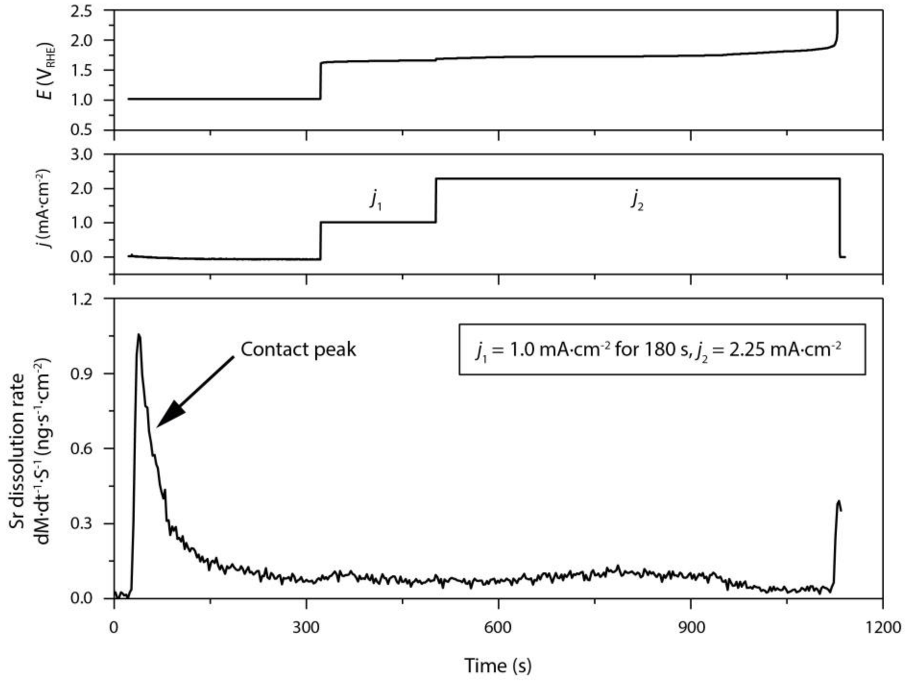

Figure S6. Chemical analysis of the electrolyte by *on-line* ICP-MS measurements. A representative dissolution measurement is shown including the region of increased Sr dissolution due to the leaching of minor Sr-rich surface phases during the first contact of the scanning flow cell with the LSCO electrode (contact peak). To investigate Sr dissolution under OER conditions, two consecutive galvanostatic holds are applied (j_1 = 1.0 mA·cm^{-2} and j_2 = 2.25 mA·cm^{-2}) while the dissolution behavior is monitored.

Fig. S6 shows the dissolution behavior of LSCO during two consecutive galvanostatic holds in analogy to Fig. 5 of the main manuscript but including the initial contact peak.

Figure S7. Angle-dependent XPS Sr 3*d*, La 3*d*$_{5/2}$ and Co 2*p*$_{3/2}$ XPS core-level spectra (Θ = 15° and Θ = 64°)

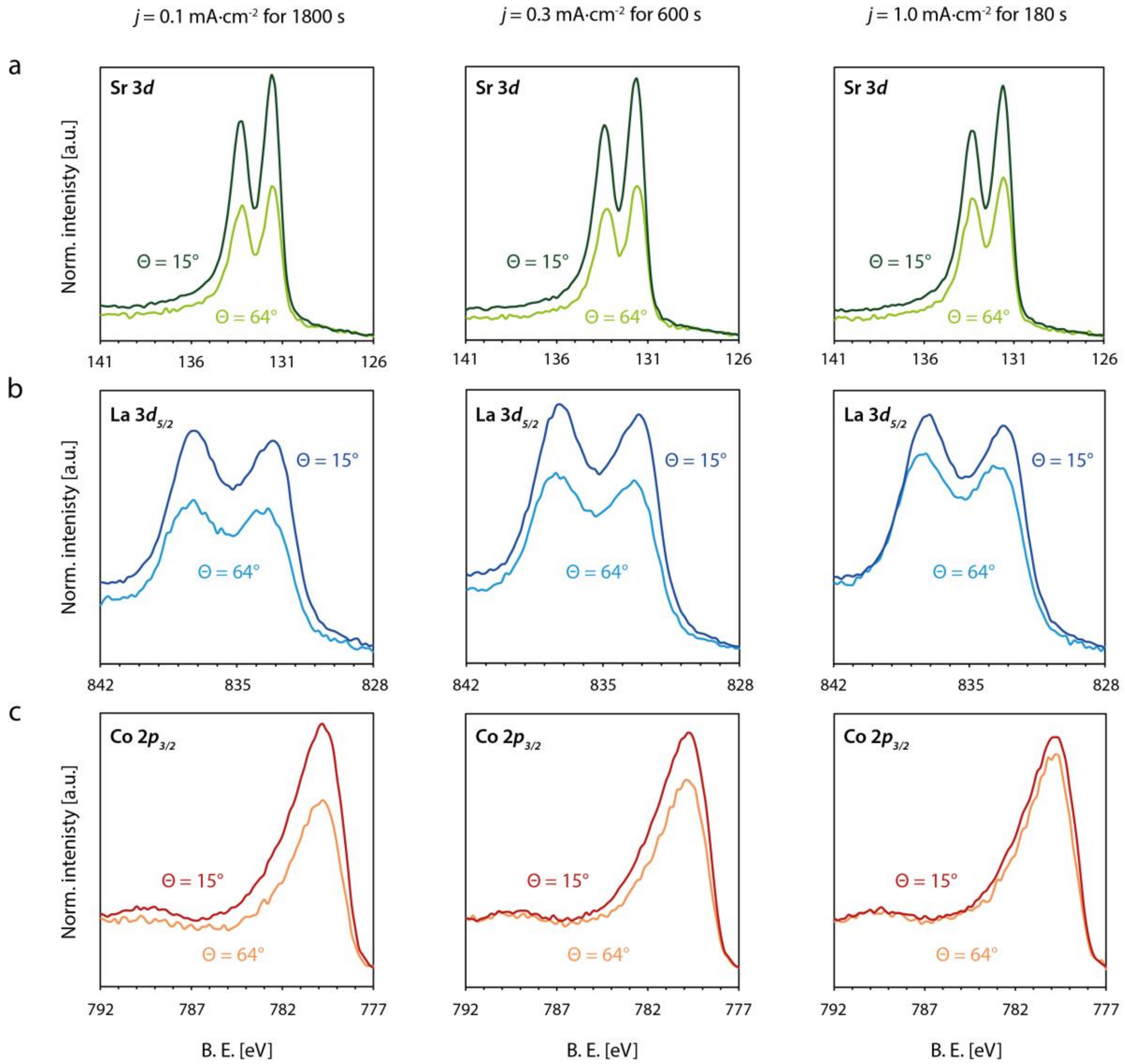

Figure S7. Sr 3*d*, La 3*d*$_{5/2}$ and Co 2*p*$_{3/2}$ XPS core-level spectra collected with a photoemission angle of Θ = 15° (less surface-sensitive) and Θ = 64° (more surface-sensitive): The data was used for the quantification of relative changes in the stoichiometry at the electrochemical interface as presented in Figure 5c in the main manuscript. All spectra are normalized to the pre-peak region.

Figure S8. Angle-dependent XPS analysis of the as-prepared LSCO.

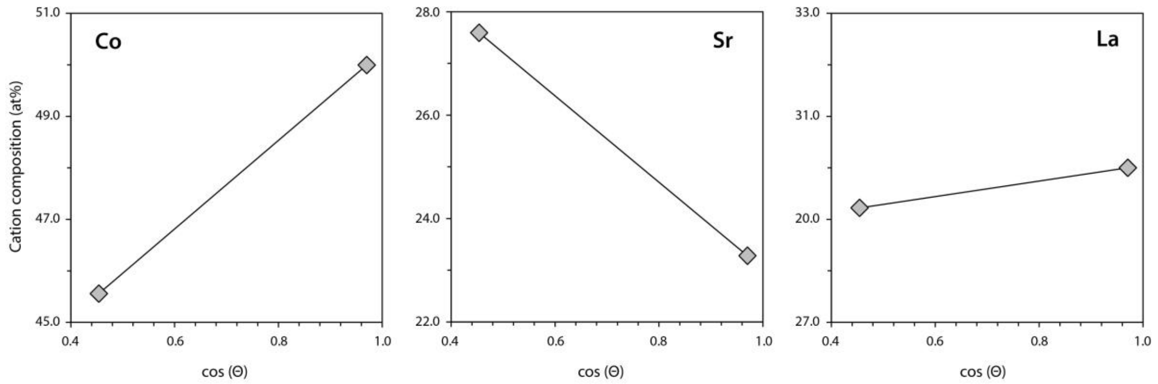

Figure S8. Angle-dependent XPS analysis of the surface stoichiometry of epitaxial LSCO electrodes in the as-prepared state. The surface is enriched by strontium and depleted from cobalt.

The as-prepared surface of the epitaxial LSCO electrodes are enriched by strontium, while cobalt is depleted from the surface. In comparison to the operated samples, discussed in the main manuscript (Fig. 5), the strontium enrichment is enhanced due to the presence of additional Sr-rich surface phases that typically dissolve during the first contact the alkaline electrolyte.

Figure S9. Surface chemistry of LSCO after the end of the catalyst lifetime.

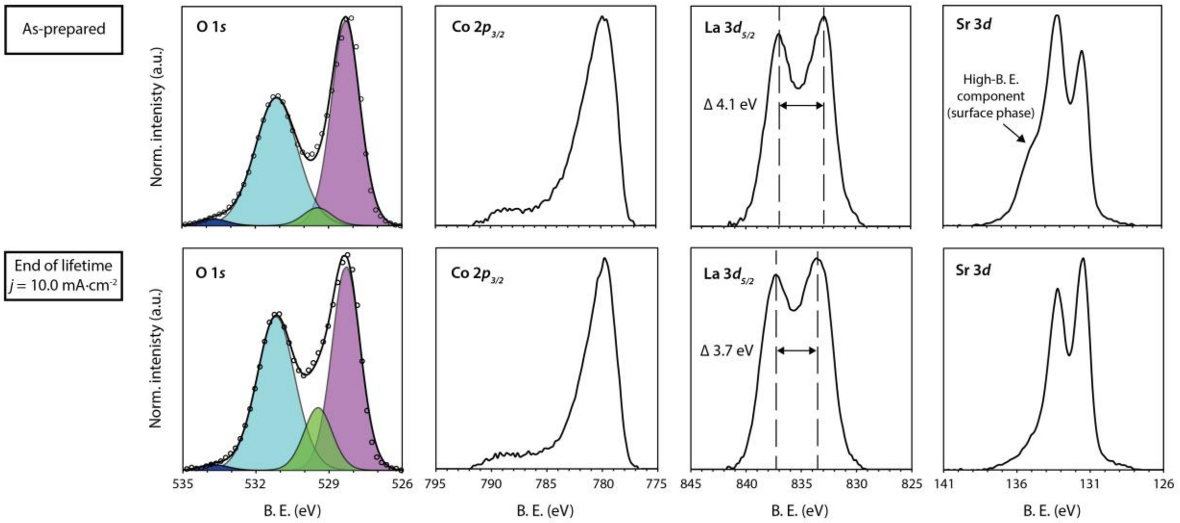

Figure S9. X-ray photoelectron spectroscopy investigations of the LSCO surface chemistry. O 1*s*, Co 2$p_{3/2}$, La 3$d_{5/2}$ and Sr 3*d* XPS core-level spectra of LSCO catalysts in the as-prepared state (first row), and after the end of lifetime after steady-state OER catalysis *j* = 10.0 mA·cm^{-2} (second row). The respective spectra are displayed for each sample state after subtraction of a Tougaard background. The O 1*s* spectra is deconvoluted by data fitting of four different components (ABO_3 lattice oxygen (purple), surface termination component (green), mixed hydroxide groups (cyan), organic components (blue). XPS analysis was performed at a photoemission angle Θ = 46°.

In contrast to the operated but active state of the LSCO electrocatalysts discussed in the main manuscript, the O 1*s* peak that can be assigned to CoO(OH) lattice oxygen has vanished after the end of lifetime is reached. Moreover, the Co 2*p* signature does not indicate the presence of a Co (III) component at the surface.

References

(1) Roddatis, V., Lole, G. & Jooss, C. In situ preparation of $Pr_{1-x}Ca_xMnO_3$ and $La_{1-x}Sr_xMnO_3$ catalysts surface for high-resolution environmental transmission electron microscopy. *Catalysts* **9**, 751 (2019).
